# Supplementary material for: The structure and diversity of strain-level variation in vaginal bacteria
Source: Microb Genom. 2021 Mar 3;7(3):mgen000543. doi: 10.1099/mgen.0.000543 (PMC8190618; doi:10.1099/mgen.0.000543)
Supplement: Supplementary material 1 [file mgen-7-0543-s001.pdf]

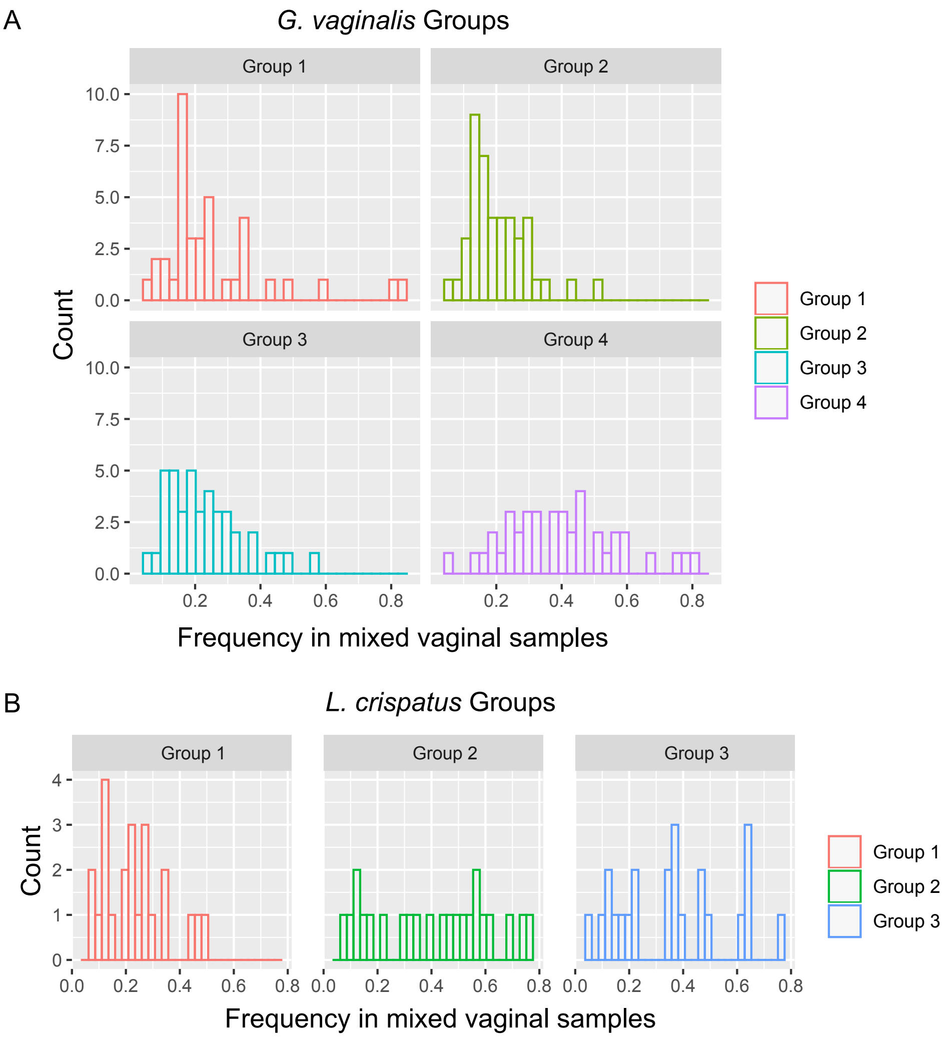

Figure S1. The relative abundance of A) *G. vaginalis* and B) *L. crispatus* groups in mixed samples based on group-specific SNPs. Each panel shows a histogram of the inferred frequency of the four *G. vaginalis* groups and three *L. crispatus* groups in vaginal samples designated as mixed populations by ADMIXTURE.
